# The complexity of vulnerability to psychosis

**DOI:** 10.1192/j.eurpsy.2021.147

**Published:** 2021-08-13

**Authors:** A. Fiorillo

**Affiliations:** Department Of Psychiatry, University of Campania “L. Vanvitelli”, Naples, Italy

**Keywords:** complexity, integrated treatments, psychosis, vulnerability

## Abstract

Schizophrenia is a complex mental disorder, which has been recently conceptualized as a neurodevelopmental disease. This conceptualization has changed the psychopathological approach to schizophrenia, which is now described as lying on a continuum from mild psychotic experiences to frank psychotic episodes. According to this theory, the presence of psychotic symptoms would represent the final pathway of a complex dysregulation and interaction of different genetic and environmental risk factors. As regards genetic liability, recent genome-wide association studies have identified a total of 108 loci containing common risk alleles, and which meet genome-wide significance. As regards environmental factors, higher rates of schizophrenia have been found in ethnic minority groups, in persons who are heavy cannabis smokers, in those who suffered from severe childhood traumas, in persons who have been reared in highly deprived settings. The identification of risk factors associated with vulnerability to psychosis is essential for improving our understanding and early detection of vulnerable individuals, and to propose tailored and timely interventions for sufferers. There is the need for an interdisciplinary approach to schizophrenia which includes screening procedures for individuals reporting specific vulnerabilities and treatment strategies tailored on patients’ needs.

**Disclosure:**

No significant relationships.

